# Addressing the Overlapping Challenges of Benign Paroxysmal Positional Vertigo (BPPV) and Persistent Postural-Perceptual Dizziness (PPPD): Impacts and Integrated Management

**DOI:** 10.7759/cureus.72019

**Published:** 2024-10-21

**Authors:** Jorge Madrigal, Francisco Zuma e Maia, Renato Cal, Bernardo F Ramos, Melissa Castillo-Bustamante

**Affiliations:** 1 Otoneurology, Centro de Vértigo y Mareo, Mexico City, MEX; 2 Otolaryngology, Clínica Maia, Canoas, BRA; 3 Otolaryngology, Para University Center (CESUPA), Belém, BRA; 4 Otolaryngology, Federal University of Espirito Santo, Vitoria, BRA; 5 School of Medicine, Health Sciences School, Universidad Pontificia Bolivariana, Medellin, COL; 6 Otolaryngology, Clinica Universitaria Bolivariana, Medellin, COL

**Keywords:** benign paroxysmal positional vertigo, persistent postural perceptual dizziness, quality of life (qol), vestibular and balance rehabilitation, vestibular disorders

## Abstract

Benign paroxysmal positional vertigo (BPPV) and persistent postural-perceptual dizziness (PPPD) are challenging vestibular disorders with overlapping symptoms that complicate diagnosis and treatment. BPPV causes transient vertigo with head movements, while PPPD involves persistent dizziness and unsteadiness. Both significantly impact the quality of life, including emotional well-being. This review examines the relationship between BPPV and PPPD, stressing the need for comprehensive, multidisciplinary management. Effective treatment must address both physical and psychological aspects, using personalized care, technological innovations, and patient education to improve outcomes and enhance the quality of life for those affected.

## Introduction and background

Benign paroxysmal positional vertigo (BPPV) and persistent postural-perceptual dizziness (PPPD) are two intricate vestibular disorders that, while distinct in nature, often intersect, leading to diagnostic and therapeutic challenges [1]. BPPV is characterized by transient vertigo episodes triggered by specific head movements, typically associated with otolithic debris dislodging within the semicircular canals [2,3]. It is a prevalent condition, particularly among the elderly, and can be effectively managed with canalith repositioning maneuvers [2,3]. In contrast, PPPD is a chronic and debilitating disorder characterized by persistent feelings of dizziness and unsteadiness, exacerbated by upright posture and movement, without any identifiable organic cause [4,5]. PPPD is often associated with heightened attention to bodily sensations and is believed to stem from maladaptive neuroplastic changes in response to a prior vestibular or non-vestibular insult [5].

Despite their distinct clinical profiles, BPPV and PPPD frequently coexist or transition into one another, complicating diagnosis and management [1,6]. For instance, patients with BPPV may develop PPPD as a consequence of prolonged avoidance behaviors due to fear of triggering vertigo, leading to deconditioning and increased sensitivity to motion [1,6]. Conversely, individuals with PPPD may experience episodic vertigo resembling BPPV, further blurring the diagnostic boundaries between the two conditions [1,6].

The interplay between BPPV and PPPD underscores the importance of a comprehensive approach to vestibular disorders, integrating diagnostic tools such as posturography, videonystagmography, and video head impulse test with multidisciplinary management strategies [7]. Understanding the nuanced relationship between these disorders is crucial for tailoring effective treatment strategies and improving outcomes in patients with vestibular dysfunction [6,7]. This narrative review aims to elucidate the complex correlation between BPPV and PPPD.

## Review

Methods

For this narrative review, a rigorous methodological approach was employed to explore the intricate correlation between BPPV and PPPD, two distinct yet potentially interrelated vestibular disorders. A comprehensive search strategy was devised to identify relevant studies, encompassing electronic databases such as PubMed, Google Scholar, and Scopus. The search strategy utilized terms including "benign paroxysmal positional vertigo," "postural perceptual persistent dizziness," and "vestibular disorders" and related terms to ensure the retrieval of pertinent literature published in English. The inclusion criteria encompassed original research articles, review papers, and clinical guidelines focusing on the pathophysiology, clinical manifestations, diagnostic modalities, and management strategies of BPPV and PPPD. The selected literature underwent meticulous screening and data extraction to capture key findings related to the epidemiology, etiology, clinical presentation, and treatment outcomes of both disorders. Special attention was given to studies elucidating potential mechanisms underlying the co-occurrence of BPPV and PPPD, as well as those investigating shared risk factors and comorbidities. Data synthesis and analysis were conducted to provide a comprehensive overview of the current understanding of BPPV and PPPD, emphasizing their overlapping features and distinct characteristics. The review also aimed to identify gaps in knowledge and propose avenues for future research to enhance the diagnosis and management of these challenging vestibular disorders. Overall, this narrative review offers a critical appraisal of the existing literature and highlights the need for a multidisciplinary approach to improve the care of patients with BPPV and PPPD.

Exploring the link: BPPV and PPPD correlation

BPPV and PPPD are two distinct vestibular disorders that can significantly impact an individual's quality of life [6,7]. BPPV is characterized by brief episodes of vertigo triggered by changes in head position, often associated with specific movements like rolling over in bed or tilting the head back [8]. On the other hand, PPPD is characterized by persistent non-vertiginous dizziness and unsteadiness, exacerbated by upright posture and movement [4]. While traditionally considered separate entities, emerging evidence suggests a complex relationship between BPPV and PPPD [1]. Some studies propose that PPPD may develop as a consequence of untreated or recurrent BPPV, highlighting the importance of recognizing and appropriately managing BPPV to prevent the development or persistence of PPPD [4,6]. Understanding the correlation between these two conditions is crucial for optimizing diagnostic and treatment strategies, ultimately improving patient outcomes and quality of life [4,6].

BPPV and PPPD: facts and controversies

Facts

BPPV and PPPD are prevalent vestibular disorders with distinct yet overlapping characteristics [1,5,9]. Age is a significant risk factor for both BPPV and PPPD, with studies indicating that the prevalence of these disorders increases with age [10,11]. BPPV is more prevalent in older adults, peaking in individuals aged 50-70 years, whereas PPPD can affect any age group but is commonly seen in middle-aged adults [10,11]. The prevalence of PPPD in children is increasing nowadays [10,11]. This age-related prevalence suggests that there may be underlying age-related changes in the vestibular system that predispose individuals to these disorders [10,11].

Gender also appears to play a role in the prevalence of BPPV and PPPD, with some studies suggesting that women may be more likely to develop these disorders than men [12]. Comorbidities, such as migraine, have also been implicated in the development of both BPPV and PPPD [13]. Migraine is a common neurological condition characterized by recurrent episodes of headache, often accompanied by sensory disturbances and nausea [13]. Studies have shown that individuals with migraine may be at an increased risk of developing vestibular disorders, including BPPV and PPPD [13]. The exact relationship between migraine and these vestibular disorders is not fully understood, but it is thought that shared pathophysiological mechanisms, such as cortical spreading depression and neurochemical imbalances, may play a role [13].

BPPV often arises spontaneously or follows head trauma, inner ear infections, or vestibular neuritis, while PPPD can develop after initial vestibular insults such as BPPV, vestibular neuritis, vestibular migraine, or traumatic brain injury, though its exact cause is not always clear [5,6]. Both disorders manifest as dizziness and unsteadiness, but their symptoms’ nature and duration vary [5,6]. Both BPPV and PPPD can significantly disrupt daily life, affecting various aspects such as work, social interactions, and overall well-being [14,15]. The sudden vertigo episodes of BPPV can be particularly debilitating, leading to difficulties in performing tasks that require head movements, such as driving or working at a computer [14]. Similarly, the persistent dizziness and unsteadiness of PPPD can make simple activities like walking or standing for long periods challenging [15]. These symptoms can also impact social interactions, as individuals may avoid social gatherings or public places due to fear of experiencing dizziness or imbalance in front of others [14,15].

Controversies

One of the primary controversies regarding PPPD involves its classification and overlap with other vestibular disorders, particularly BPPV [6]. Some researchers consider PPPD to be a distinct clinical entity, while others suggest it may be a consequence or manifestation of an underlying vestibular pathology like BPPV [15,16]. This overlap often complicates diagnosis and management, as patients may exhibit symptoms indicative of both disorders [6,14]. Additionally, while the diagnostic criteria specify that symptoms are not better accounted for by another disease or disorder, the same Bárány Society paper acknowledges that PPPD may coexist with other diseases or disorders. This ambiguity highlights the complexity of identifying and managing PPPD. The role of vestibular rehabilitation in managing these disorders is a subject of debate [16-18]. While it is a recognized and effective treatment for BPPV, its effectiveness for PPPD is a matter of varying perspectives [19]. Some studies indicate that vestibular rehabilitation can be advantageous for PPPD, as it may aid in central compensation and enhance postural control [20]. However, contrasting views suggest that it might not adequately target the underlying maladaptive central processing that contributes to PPPD [21,22].

BPPV and PPPD challenges and quality of life

When BPPV and PPPD coexist or overlap in a patient, several challenges can arise in terms of diagnosis and management [1,6]. These challenges stem from the complex and overlapping nature of the two disorders, as well as their distinct clinical presentations and treatment approaches [1,6]. One of the main challenges is distinguishing between the two disorders, as they can present with similar symptoms such as dizziness and unsteadiness [4,6]. The overlap in symptoms can make it difficult to differentiate between the two disorders based on clinical presentation alone [1]. BPPV is characterized by brief, intense episodes of vertigo triggered by specific head movements, often associated with positional nystagmus [9]. In contrast, PPPD manifests as a persistent sensation of non-vertiginous dizziness and unsteadiness, exacerbated by upright posture and movement, without positional nystagmus [23]. To differentiate between these disorders, a comprehensive evaluation is essential [9,24]. History-taking should focus on symptom duration and triggers, while physical examination should include positional testing and assessment of eye movements [25]. To strengthen the differential diagnosis between PPPD and other vestibular disorders, including bilateral vestibular hypofunction, the video head impulse test (vHIT) can be valuable [26]. Unlike PPPD, which does not typically present with measurable peripheral vestibular deficits, bilateral vestibular hypofunction is characterized by reduced vestibulo-ocular reflex gains on vHIT [26]. Including vHIT as part of the diagnostic workup helps to identify or rule out underlying peripheral vestibular dysfunction, refining the diagnostic process for patients who may present with overlapping symptoms across these disorders [26]. Additionally, a neurological examination is crucial to rule out central causes of dizziness [25]. Psychological assessment is also important, as PPPD is often associated with anxiety and somatic symptom disorder [27]. Treatment response can further aid in differentiation, with BPPV typically responding well to repositioning maneuvers and PPPD requiring a multidisciplinary approach involving vestibular rehabilitation and cognitive behavioral therapy [19,28].

Impact on quality of life

The impact of BPPV and PPPD on quality of life is profound, affecting various aspects of daily functioning and well-being [15,29]. BPPV, characterized by sudden episodes of vertigo triggered by changes in head position, can lead to significant limitations in activities of daily living [30]. Patients often report feeling anxious or fearful of triggering vertigo, which can restrict their mobility and independence [31]. The recurrent nature of BPPV episodes can also cause frustration and a sense of unpredictability, further impacting quality of life [31].

On the other hand, PPPD, characterized by persistent feelings of dizziness and unsteadiness unrelated to head movements, can have a debilitating effect on the quality of life [28]. Patients with PPPD often report difficulty with balance, spatial orientation, and concentration, which can interfere with work, social activities, and leisure pursuits [32]. The persistent nature of PPPD symptoms can lead to feelings of frustration, isolation, and depression, further exacerbating the impact on quality of life [28]. Both BPPV and PPPD can also have indirect effects on quality of life, such as sleep disturbances, fatigue, and limitations in social interactions [15,28,33]. The emotional toll of living with these chronic vestibular disorders should not be underestimated, as patients often experience anxiety, depression, and a sense of loss of control over their lives [28,34].

The emotional toll of living with chronic vestibular disorders like PPPD is profound and multifaceted, often overshadowing the physical symptoms themselves [6,15]. The emotional dimensions of various questionnaires should be mentioned in a narrative review, taking into consideration specific scales such as the Nigata and the emotional component of the Dizziness Handicap Inventory (DHI). Additionally, the Visual Vertigo Analogue Scale can aid in differentiating BPPV from PPPD and even healthy individuals, as already published. BPPV is generally considered an episodic rather than a chronic vestibular disorder due to its often-transient nature and the availability of effective repositioning maneuvers [9]. However, if BPPV remains undiagnosed or untreated, symptoms can persist indefinitely, leading to recurrent vertigo episodes that significantly impair the quality of life [14]. Prolonged symptoms can contribute to chronic dizziness, increased fall risk, and substantial psychological distress, as patients may experience heightened anxiety and limitations in daily activities [14]. This underscores the importance of early and accurate diagnosis, as prompt identification and management of BPPV can prevent the progression to chronic symptomatology [14]. Timely treatment reduces the long-term impact on vestibular function and mitigates the broader psychosocial effects of prolonged dizziness, improving both physical well-being and overall quality of life for affected individuals [14]. Furthermore, the roles of selective serotonin reuptake inhibitors (SSRIs) and betahistine in the management of both BPPV and PPPD warrant discussion, as they may offer therapeutic benefits in addressing the associated emotional and vestibular symptoms [14].

Patients frequently experience a pervasive sense of anxiety, as the unpredictable nature of vertigo attacks or persistent dizziness leaves them constantly on edge, fearing when the next episode might occur [1,6,15,28]. This anxiety can lead to hypervigilance and avoidance behaviors, where individuals might limit their activities, avoid certain movements, or stay away from busy environments, further isolating themselves [1,6,15,28].

Depression is another common emotional consequence. The chronic, relentless nature of these vestibular disorders can lead to a sense of hopelessness and despair [35-37]. Patients may feel trapped in their condition, perceiving a bleak outlook on recovery or improvement [35-37]. The constant battle with symptoms can drain their emotional resilience, leaving them feeling fatigued and disheartened [35-37]. Social withdrawal is a frequent byproduct, as the fear of experiencing symptoms in public or during social interactions can lead to decreased participation in social activities, exacerbating feelings of loneliness and isolation [38]. Moreover, the cognitive load of managing these vestibular disorders adds to the emotional burden [36]. The need to constantly monitor symptoms, avoid triggers, and manage treatment regimens can become overwhelming [36]. This constant vigilance can impair cognitive functions such as memory, attention, and executive functioning, further contributing to feelings of inadequacy and frustration [39]. Patients often report a diminished sense of self-efficacy and control over their lives, which can severely impact their overall mental health and well-being [39].

The emotional distress associated with vestibular disorders such as BPPV and PPPD is not just a secondary consequence but a core component of the disease burden [6,15]. Effective management must, therefore, include psychological support, such as counseling or cognitive-behavioral therapy, to help patients develop coping strategies, address anxiety and depression, and rebuild their confidence and quality of life [40]. Integrative approaches that address both the physical and emotional aspects of these disorders are crucial for comprehensive care and improved patient outcomes [40]. Some studies have suggested that patients with BPPV who also experience anxiety tend to report greater complaints of residual dizziness following successful repositioning maneuvers [41-44]. This phenomenon may reflect the interplay between psychological factors and vestibular symptoms, indicating that anxiety can amplify patients' perceptions of dizziness even when the underlying mechanical issue has been resolved [41-44]. These findings highlight the importance of considering psychological well-being in the management of BPPV, as addressing anxiety may be crucial for improving treatment outcomes and enhancing overall patient satisfaction [41-44]. Furthermore, understanding the relationship between anxiety and residual dizziness can inform the development of more comprehensive treatment approaches that include psychological support alongside vestibular rehabilitation.

Future directions in the management of BPPV and PPPD aim to refine treatment approaches and enhance patient outcomes [41,45]. Personalized treatment strategies based on patient-specific factors such as age, comorbidities, and treatment response could improve the efficacy of interventions [46]. Integrating technology like virtual reality and mobile applications may enhance vestibular rehabilitation and provide remote support [46]. Longitudinal studies are needed to understand the natural course and prognostic factors of these disorders, and patient education and empowerment programs can help patients better manage their symptoms [47]. Advocacy for better recognition of vestibular disorders in healthcare policy and global collaboration can further improve care for BPPV and PPPD, ultimately enhancing the quality of life for affected individuals [15,46,47].

## Conclusions

BPPV and PPPD pose significant diagnostic and therapeutic challenges due to their overlapping symptoms. These disorders greatly impact the quality of life, affecting both physical and emotional well-being. Effective management requires a multidisciplinary approach that addresses both aspects. Future strategies should focus on personalized care, technological integration, and improved patient education to enhance outcomes for those affected by BPPV and PPPD.

## References

[1] Casani AP, Ducci N, Lazzerini F, Vernassa N, Bruschini L (2023). Preceding benign paroxysmal positional vertigo as a trigger for persistent postural-perceptual dizziness: which clinical predictors?. Audiol Res.

[2] von Brevern M, Bertholon P, Brandt T, Fife T, Imai T, Nuti D, Newman-Toker D (2015). Benign paroxysmal positional vertigo: diagnostic criteria. J Vestib Res.

[3] Imai T, Inohara H (2022). Benign paroxysmal positional vertigo. Auris Nasus Larynx.

[4] Knight B, Bermudez F, Shermetaro C (2024). Persistent postural-perceptual dizziness. StatPearls [Internet].

[5] Staab JP, Eckhardt-Henn A, Horii A, Jacob R, Strupp M, Brandt T, Bronstein A (2017). Diagnostic criteria for persistent postural-perceptual dizziness (PPPD): consensus document of the committee for the Classification of Vestibular Disorders of the Bárány Society. J Vestib Res.

[6] Gambacorta V, D'Orazio A, Pugliese V, Di Giovanni A, Ricci G, Faralli M (2022). Persistent postural perceptual dizziness in episodic vestibular disorders. Audiol Res.

[7] Oka M, Ichijo K, Koda K (2023). Preceding balance disorders affect vestibular function in persistent postural-perceptual dizziness. J Clin Med.

[8] Lee SH, Kim JS (2010). Benign paroxysmal positional vertigo. J Clin Neurol.

[9] Palmeri R, Kumar A (2024). Benign paroxysmal positional vertigo. StatPearls [Internet].

[10] Zhang L, Jiang W, Tang L, Liu H, Li F (2022). Older patients with persistent postural-perceptual dizziness exhibit fewer emotional disorders and lower vertigo scores. Sci Rep.

[11] Wassermann A, Finn S, Axer H (2022). Age-associated characteristics of patients with chronic dizziness and vertigo. J Geriatr Psychiatry Neurol.

[12] Trinidade A, Harman P, Stone J, Staab JP, Goebel JA (2020). Assessment of potential risk factors for the development of persistent postural-perceptual dizziness: a case-control pilot study. Front Neurol.

[13] Çakır S, Sahin A, Gedik-Soyuyuce O, Gence Gumus Z, Sertdemir İ, Korkut N, Yalınay Dikmen P (2024). Assessing the impact of migraine on benign paroxysmal positional vertigo symptoms and recovery. BMC Neurol.

[14] Handa PR, Kuhn AM, Cunha F, Schaffleln R, Ganança FF (2005). Quality of life in patients with benign paroxysmal positional vertigo and/or Ménière's disease. Braz J Otorhinolaryngol.

[15] Steensnaes MH, Knapstad MK, Goplen FK, Berge JE (2023). Persistent Postural-Perceptual Dizziness (PPPD) and quality of life: a cross-sectional study. Eur Arch Otorhinolaryngol.

[16] Azami M, Fushiki H, Tsunoda R, Kamo T, Ogihara H, Tanaka R, Kato T (2023). Clinical features of persistent postural-perceptual dizziness with isolated otolith dysfunction as revealed by VEMP and vHIT findings. Front Neurol.

[17] Bressi F, Vella P, Casale M (2017). Vestibular rehabilitation in benign paroxysmal positional vertigo: reality or fiction?. Int J Immunopathol Pharmacol.

[18] Popkirov S, Stone J, Holle-Lee D (2018). Treatment of persistent postural-perceptual dizziness (PPPD) and related disorders. Curr Treat Options Neurol.

[19] Nada EH, Ibraheem OA, Hassaan MR (2019). Vestibular rehabilitation therapy outcomes in patients with persistent postural-perceptual dizziness. Ann Otol Rhinol Laryngol.

[20] Ata G, Şakul AA, Kılıç G, Çelikyurt C (2023). Comparison of the efficacy of vestibular rehabilitation and pharmacological treatment in benign paroxysmal positional vertigo. Indian J Otolaryngol Head Neck Surg.

[21] Herdman D, Norton S, Murdin L, Frost K, Pavlou M, Moss-Morris R (2022). The INVEST trial: a randomised feasibility trial of psychologically informed vestibular rehabilitation versus current gold standard physiotherapy for people with Persistent Postural Perceptual Dizziness. J Neurol.

[22] Fujimoto C, Oka M, Ichijo K (2023). The effect of self-management vestibular rehabilitation on persistent postural-perceptual dizziness. Laryngoscope Investig Otolaryngol.

[23] Riccelli R, Passamonti L, Toschi N (2017). Altered insular and occipital responses to simulated vertical self-motion in patients with persistent postural-perceptual dizziness. Front Neurol.

[24] You P, Instrum R, Parnes L (2019). Benign paroxysmal positional vertigo. Laryngoscope Investig Otolaryngol.

[25] Choi WY, Gold DR (2019). Vestibular disorders: pearls and pitfalls. Semin Neurol.

[26] Judge PD, Janky KL, Barin K (2017). Can the video head impulse test define severity of bilateral vestibular hypofunction?. Otol Neurotol.

[27] Zang J, Zheng M, Chu H, Yang X (2024). Additional cognitive behavior therapy for persistent postural-perceptual dizziness: a meta-analysis. Braz J Otorhinolaryngol.

[28] Vats AK, Kothari S, Biswas A (2023). Vestibular rehabilitation of the persons affected by benign paroxysmal positional vertigo (BPPV) by physical therapy and repositioning maneuvers. Ann Indian Acad Neurol.

[29] Teh CS, Prepageran N (2022). The impact of disease duration in persistent postural-perceptual dizziness (PPPD) on the quality of life, dizziness handicap and mental health. J Vestib Res.

[30] Kourosh P (2014). Benign paroxysmal positional vertigo: an integrated perspective. Adv in Otol.

[31] Shu Y, Liao N, Fang F, Shi Q, Yan N, Hu Y (2023). The relationship between psychological conditions and recurrence of benign paroxysmal positional vertigo: a retrospective cohort study. BMC Neurol.

[32] Castro P, Bancroft MJ, Arshad Q, Kaski D (2022). Persistent postural-perceptual dizziness (PPPD) from brain imaging to behaviour and perception. Brain Sci.

[33] Smith LJ, Wilkinson D, Bodani M, Surenthiran SS (2024). Cognition in vestibular disorders: state of the field, challenges, and priorities for the future. Front Neurol.

[34] Mutlu B, Topcu MT (2022). Investigation of the relationship between vestibular disorders and sleep disturbance. Int Arch Otorhinolaryngol.

[35] Hsu CL, Tsai SJ, Shen CC, Lu T, Hung YM, Hu LY (2019). Risk of benign paroxysmal positional vertigo in patients with depressive disorders: a nationwide population-based cohort study. BMJ Open.

[36] Smith LJ, Pyke W, Fowler R, Matthes B, de Goederen E, Surenthiran S (2024). Impact and experiences of vestibular disorders and psychological distress: qualitative findings from patients, family members and healthcare professionals. Health Expect.

[37] Wurthmann S, Holle D, Obermann M (2021). Reduced vestibular perception thresholds in persistent postural-perceptual dizziness - a cross-sectional study. BMC Neurol.

[38] Quach LT, Burr JA (2021). Perceived social isolation, social disconnectedness and falls: the mediating role of depression. Aging Ment Health.

[39] Bhattacharyya R, Barman A, Antony F (2024). Influence of BPPV and Meniere's disease on cognitive abilities: a questionnaire-based study. J Otol.

[40] Waterston J, Chen L, Mahony K, Gencarelli J, Stuart G (2021). Persistent postural-perceptual dizziness: precipitating conditions, co-morbidities and treatment with cognitive behavioral therapy. Front Neurol.

[41] Axer H, Finn S, Wassermann A, Guntinas-Lichius O, Klingner CM, Witte OW (2020). Multimodal treatment of persistent postural-perceptual dizziness. Brain Behav.

[42] Özgirgin ON, Kingma H, Manzari L, Lacour M (2024). Residual dizziness after BPPV management: exploring pathophysiology and treatment beyond canalith repositioning maneuvers. Front Neurol.

[43] Teggi R, Giordano L, Bondi S, Fabiano B, Bussi M (2011). Residual dizziness after successful repositioning maneuvers for idiopathic benign paroxysmal positional vertigo in the elderly. Eur Arch Otorhinolaryngol.

[44] Seok JI, Lee HM, Yoo JH, Lee DK (2008). Residual dizziness after successful repositioning treatment in patients with benign paroxysmal positional vertigo. J Clin Neurol.

[45] Kouijzer MM, Kip H, Bouman YH, Kelders SM (2023). Implementation of virtual reality in healthcare: a scoping review on the implementation process of virtual reality in various healthcare settings. Implement Sci Commun.

[46] Guralnik JM, Kritchevsky SB (2010). Translating research to promote healthy aging: the complementary role of longitudinal studies and clinical trials. J Am Geriatr Soc.

[47] Bhattacharyya N, Gubbels SP, Schwartz SR (2017). Clinical practice guideline: benign paroxysmal positional vertigo (update). Otolaryngol Head Neck Surg.

